# Increased prevalence of hypertension among people living with HIV: where to begin?

**DOI:** 10.1590/0037-8682-0564-2019

**Published:** 2020-09-11

**Authors:** Aldrey Nascimento Costa, Fernando Val, Álvaro Elias Macedo, Nadia Cubas-Vega, Paola López Del Tejo, Marly M. Marques, Aristóteles Comte de Alencar, Marcus Vinicius Guimarães de Lacerda

**Affiliations:** 1 Universidade do Estado do Amazonas, Escola Superior de Ciências da Saúde, Manaus, AM, Brasil.; 2 Universidade Federal do Amazonas, Manaus, AM, Brasil.; 3 Fundação de Medicina Tropical Doutor Heitor Vieira Dourado, Manaus, AM, Brasil.; 4 Instituto Leônidas & Maria Deane, Fundação Oswaldo Cruz, Manaus, AM, Brasil.

**Keywords:** HIV, Non-communicable diseases, Cardiovascular diseases, hypertension

## Abstract

**INTRODUCTION:**

Cardiovascular diseases (CDVs) have become increasingly important for progressively older people living with HIV (PLHIV). Identification of gaps requiring improvement in the care cascade for hypertension, a primary risk factor for CVDs, is of utmost importance. This study analyzed the prevalence of hypertensive status and described the care cascade for hypertension screening, diagnosis, treatment, treatment adherence, and management in PLHIV.

**METHODS:**

This cross-sectional study included 298 PLHIV (age >40 years) who visited a referral center in the western Brazilian Amazon. Data were collected through a structured questionnaire interview and medical examinations. Thus, information regarding sociodemographic and clinical aspects, blood pressure, weight, height, body mass index, and laboratory profile was obtained. Descriptive and analytical statistics were performed, and results were considered significant ifp <0.05.

**RESULTS:**

In total, 132 (44.3%) participants reported that their blood pressure was never measured. The prevalence of hypertension was found to be 35.9% (107/298). Of these 107 participants, only 36 (33.6%) had prior knowledge of their hypertensive status, and 19 of 36 (52.7%) participants had visited a physician or cardiologist to seek treatment. Adherence to the BP-lowering treatment was noted in 11 (10.2%) participants.

**CONCLUSIONS:**

An increased prevalence of hypertension was found, and most of the hypertensive participants were unaware of their hypertensive status. In addition, blood pressure control was poor in the study population. This indicated that public health professionals did not sufficiently consider the full spectrum of healthcare and disease management for PLHIV.

## INTRODUCTION

In 2018, the Joint United Nations Programme on HIV/AIDS (UNAIDS) estimated that of approximately 38 million people living with HIV (PLHIV) worldwide, 2.2 million were from Latin America and the Caribbean region^1^.

The number of PLHIV in Brazil has increased^2^, despite the efforts and actions taken in recent years to prevent and treat HIV^3^ ^-^ ^5^. According to the Brazilian Notification Disease Information System (*Sistema de Informação de Agravos de Notificação* [SINAN]), the northern region of the country has the highest national rate of newly-diagnosed cases^2^. This rate is even more significant, considering the difference in population density throughout Brazil. In 2010, the demographic density of the northern region was 4.12, whereas that of the South East region was 86.9^6^.

Increased worldwide coverage of highly active antiretroviral therapy (HAART) has improved patient management and assisted in reducing AIDS mortality; thus, the life expectancy of PLHIV has increased^1^. However, the prevalence of cardiovascular diseases (CVDs) and CVD-related mortality has increased among PLHIV, who are up to two times more likely to present a CVD episode compared with HIV-negative individuals^7^ ^-^ ^9^. The pathogenesis of CVD, including hypertension, in PLHIV is a complex interaction between traditional risk factors (dyslipidemia, diabetes, smoking, and sedentary lifestyle), HIV infection (viral activity, decreased TCD4+ cell count, chronic systemic inflammation, and endothelial dysfunction), and risk factors associated with HAART (dyslipidemia, endothelial damage, carotid thickness, and lipodystrophy)^10^ ^-^ ^12^.

The “cascade of care” concept aids in portraying and analyzing patient behavior regarding the diagnosis, treatment, retention steps, and achievement of predefined goals in HIV care, especially achieving UNAIDS 90-90-90 targets by 2020. The UNAIDS 90-90-90 targets are as follows: to diagnose 90% of all PLHIV worldwide, provide antiretroviral therapy to 90% of those diagnosed, and achieve viral load suppression in 90% of those treated^13^. However, room for improvement in each stage exists. This is true for blood pressure (BP) control. Recent data shows that the major cause of uncontrolled BP is lack of awareness followed by an absence of treatment^14^.

Free health care is provided to all Brazilians and foreign residents in Brazil through a unified health care system (*Sistema Único de Saúde* [SUS]). Health care includes care for hypertension, which occurs at the primary care level, and for infectious diseases such as HIV, which occurs at the tertiary care level. Despite free access to medical care for both diseases, overlapping situations remain a matter of concern and should be addressed urgently. Currently, no treatment strategies that are proven to effectively prevent CVDs in PLHIV are available. Therefore, because CVDs are becoming increasingly important from a public health perspective for progressively older PLHIV, it is of utmost importance to identify stages in the care cascade that require improvement. Therefore, this study analyzed the prevalence of hypertensive status among PLHIV who attended routine follow-up consultations at a referral outpatient care clinic for infectious diseases in the western Brazilian Amazon, described the hypertension care cascade, and identified possible points of intervention to better address the condition in PLHIV.

## METHODS

### Study design and ethical considerations

We conducted a cross-sectional study at an HIV/AIDS outpatient clinic in Manaus, Amazonas state, western Brazilian Amazon. The HIV/AIDS outpatient care clinic at the *Fundação de Medicina Tropical Doutor Heitor Vieira Dourado*, is a tertiary care referral center for infectious diseases. This study was approved by the local Ethical Review Board (approval number: CAAE 76111617.6.0000.0005) and was conducted in compliance with the principles of good clinical practice.

### Study participants and data collection

The study was conducted between June 2018 and September 2018. The study population comprised a convenience sample of PLHIV, men and women (age >40 years), who were on antiretroviral treatment, and visited the outpatient clinic for follow-ups. Individuals were invited to participate in the study as they waited for the routine consultation with the infectious disease physician. Individuals who agreed to the study procedures and signed the informed consent form were included in the study. All the participants had their BP measured, self-reported ethnic origin, and received a structured questionnaire interview on sociodemographic data, family income, lifestyle habits, medical history, and medication use. Anthropometric characteristics (height and weight) were verified by the medical team. Height without shoes was measured in centimeters using a stadiometer, and weight, with light clothing and without shoes, was measured in kilograms on a mechanical weighing scale. The body mass index (BMI) was calculated using the measured weight and height. The laboratory profile (total and partial cholesterol, triglycerides, glycemia, creatinine, CD4+ lymphocytes, CD8+ lymphocytes, and viral load) and HIV treatment history were obtained from the hospital’s electronic medical chart.

### Study definitions

In our study, hypertension was defined, according to the Brazilian Society of Cardiology^15^, as systolic BP (SBP) ≥140 mmHg or diastolic BP (DBP) ≥90 mmHg**.** Normal BP was defined as SBP ≤139 mmHg or DBP ≤89 mmHg. A calibrated digital sphygmomanometer was used to measure each participant’s BP. The BP was measured twice, with a 15-minute interval between the two measurements. Participants were asked to quietly sit in an isolated room for 10 minutes before the measurements. To avoid transient elevation in BP secondary to the white-coat syndrome, participants were instructed to be accompanied by a person of their choice during the measurements. BP was independently measured by two researchers. Participants with a history of hypertension were classified as hypertensive, although their BP was within the normal range during the measurement. All the participants, including those classified as normotensive, were asked whether they used BP-lowering medication to avoid classifying them as normotensive.

Further, a structured questionnaire was applied to verify, graphically construct, and analyze the levels of the care cascade received by the participants. The definitions of these levels are described in Table 1.

**TABLE 1:** Definitions of the levels of the hypertension care cascade.

Level	Definition
	
Awareness	The patient had been previously informed of their hypertension status by a health professional
Care	The patient had visited an outpatient clinic at least twice in the previous year and was seen by a physician or cardiologist for hypertension treatment
Treatment	The patient had already been prescribed blood pressure-lowering drugs
Adherence	The patient referred to taking blood pressure-lowering drugs as prescribed by the physician

Definitions adapted from Prenissi et al. 2019^26^ and Wozniak et al. 2016^27^.

### Statistical analysis

Descriptive statistics were used for demographic and clinical data. Independent t-tests or Wilcoxon-Mann-Whitney and chi-square (χ2) tests were performed to compare groups stratified by two outcomes: normotensive or hypertensive. Continuous variables with normal distribution are described as means and standard deviation and those without normal distribution as medians and interquartile range (IQR). The normality was verified using the Shapiro-Wilk test. The level of confidence was set at 95%. Statistical significance was defined as p <0.05. All statistical analyses were performed using Stata v13 (Stata Corp, College Station, Texas, USA).

## RESULTS

This study included 298 PLHIV (men: 215 [72.2%]). The median age of the study population was 52 (IQR: 45-57) years. Further demographic characteristics are presented in Table 2.

**TABLE 2:** Demographic characteristics of the study population.

Variables	Normotensive	Hypertensive	p-value	All
	(n = 191)	(n = 107)		(N = 298)
**Sex**				
Male n (%)	137 (71.7)	78 (72.9)	0.829	215 (72.2)
Female n (%)	54 (28.2)	29 (27.1)		83 (27.8)
Sexual orientation*				
Heterosexual n (%) l	120 (62.8)	62 (57.9)	0.563	182 (61.1)
Homosexual n (%)	45 (23.5)	31 (28.9)		76 (25.5)
Bisexual n (%)	4 (2.2)	4 (3.8)		8 (2.68)
**Age (years)** median (IQR)	50 (43-56)	53 (48-60)	0.001	52 (45-57)
**Ethnic Group**				
White n (%)	26 (13.6)	11 (10.2)	0.043	37 (12.4)
Black n (%)	19 (9.6)	3 (2.8)		22 (7.38)
Latino or Hispanic n (%)	146 (76.4)	93 (86.9)		239 (80.2)
**Income (R$)** ^#^ median (IQR)	1000 (950-2000)	1200 (900-2100)	0.619	1092 (900-2000)
**Life habits**				
Smoking ^$^ n (%)	52 (27.2)	14 (13)	0.005	66 (22.1)
Alcohol abuse n (%)	77 (40.3)	49 (45.8)	0.398	126 (42.3)
**Family history of hypertension** ^**^ n (%)	121 (63.3)	69 (64.4)	0.513	190 (63.7)
**BMI** median (IQR)	26 (22.7-28.7)	27 (24-30)	0.008	26 (23-29)
**Triglycerides (mg/dL)** median (IQR)	156 (117-213)	160 (117-243)	0.494	158 (117-222)
**LDL cholesterol (mg/dL)** median (IQR)	103 (83-128)	118 (89-142)	0.019	107 (85-135)
**Time to HIV diagnosis (years)** median (IQR)	8 (4-12.5)	8 (5-13)	0.701	8 (4-13)
**Duration of HIV treatment (years)** median (IQR)	5.5 (3-9)	7 (4-9)	0.234	6 (0-9)
**Nadir CD4+** median (IQR)	235 (109.5-389)	272 (119-448)	0.341	251 (111-420)
**CD4+ cell/µL** median (IQR)	555 (395-787)	610 (426-916)	0.138	571 (410-800)
**Viral load** median (IQR)	0 (0-0)	0 (0-0)	0.969	0 (0-0)

**Abbreviations: BMI:** body mass index; **CD:** cluster of differentiation; **cm:** centimeters; **dL:** deciliter; **HIV:** human immunodeficiency virus; **LDL:** low-density lipoprotein; **mg:** milligrams; **R$:** Reais, Brazilian currency; **y:** years. *32 participants did not declare sexual orientation; ^#^32 participants did not declare income; ^$^3 participants did not declare smoking habits; **66 participants did not know their previous family history of hypertension.

The BMI was higher in the hypertensive group than in the normotensive group (p = 0.008). Among 298 participants, 66 (22.1%) were ex-smokers. In total, 52 (78.8%) of 66 ex-smokers were classified into the normotensive group and 14 (21.2%) into hypertensive group (p = 0.005). The plasma level of low-density lipoprotein (LDL) cholesterol was significantly higher in the hypertensive group than in the normotensive group (118 mg/dL versus 103 mg/dL, p = 0.019). A total of 260 (87.2%) participants had an undetectable viral load. The median CD4+ count was 571 cells/µL (range: 410-800 cells/µL).

Duration and adherence to HAART varied among study participants. The median treatment time was 6 years (range: 0-27 years), and 256 of 298 (85.9%) participants had a treatment adherence greater than 90%.

### Hypertension care cascade

**FIGURE 1:**
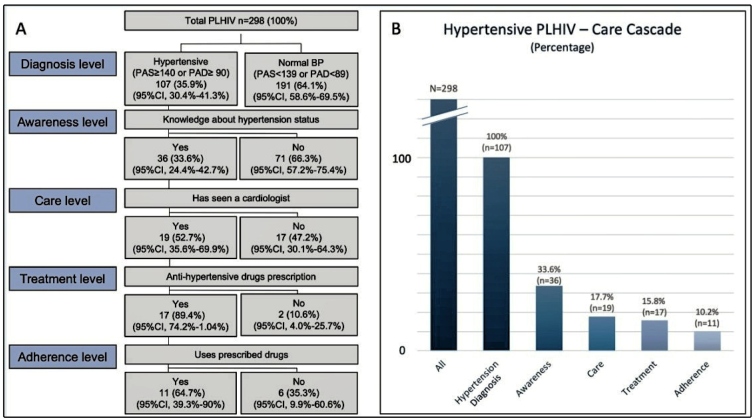
Hypertension care cascade for PLHIV: **(A)** flowchart and **(B)** bar graph.

A total of 132 (44.3%) participants reported that they had never measured their BP. In total, 191 (64.1%) participants were normotensive (SBP ≤ 139 mmHg or DBP ≤ 89 mmHg) and 107 (35.9%) participants presented signs of systemic hypertension. Among 107 (35.9%) participants diagnosed with hypertension, only 36 (33.6%) had prior knowledge of their hypertensive status and 11 (10.2%) reported adherence to their prescribed BP-lowering treatment. Slightly more than half of these participants (19/36 [52.7%]) had visited a cardiologist or physician to seek treatment, and 17 (89.4%) of these 19 participants had already been prescribed medication. A total of 11 (64.7%) participants who had been prescribed BP-lowering medication presented treatment adherence (Figure 1A and Figure 1B).

## DISCUSSION

This study revealed that a high proportion of PLHIV who were more than 40 years old had elevated BP. It is estimated that hypertension affects 36 million (32.5%) individuals in Brazil^15^. The prevalence of hypertensive status among PLHIV in this study was higher than that in the study by Cunha et al.^16^. Cunha et al. reported that the prevalence of hypertension among HIV outpatients was 17.3%.

Population characteristics and modifiable CVD risk factors may be associated with the hypertensive status among PLHIV. An African study reported that male sex is a significant factor associated with hypertension^17^. However, male sex was not found to be associated with hypertension in our study. In contrast, the age of the study population was significantly associated with the hypertensive status in the African study as well as in our study. In our study, the median BMI was high, indicating that participants had excess body weight. Being overweight is a modifiable risk factor that was found to be associated with hypertension in the current study, which corroborates with the findings of a study conducted in Poland and another in Nigeria^17^ ^,^ ^18^. Smoking, another modifiable CVD risk factor, was found to be significantly associated with the hypertensive status in our study population. However, smoking habits were not reported to be associated with hypertension in PLHIV in Norway^19^. In Brazil, 9.3% of individuals aged more than 18 years have smoking habits; however, the prevalence is lower (6.4%) in Manaus^20^. Other factors, such as social, financial, and cultural characteristics of both countries may explain this discrepancy.

Elevated LDL cholesterol level is an established risk factor for atherosclerotic CVD. Strategies to the lower LDL cholesterol level include the use of statins and exercise. Both the strategies have been reported to reported to positively enhance lipid and immune status and and decrease the level of CVD markers in such a population^21^. More than half of the study participants had a high LDL cholesterol level, with hypertensive individuals presenting higher levels. Elevated LDL cholesterol levels have been widely associated with increased CVD risk in PLHIV, although not all populations have a high prevalence of hypertension or an association with HIV^22^ ^,^ ^23^.

A steep decline was observed in several levels of the care cascade for controlling hypertension in the study population. This reflects a failure of primary health care, although several difficulties could be hampering timely hypertension diagnosis and management in this population. The failure points include the requirement for timely change in well-established risk factors in the primary care approach and the lack of early detection of hypertension. This could be explained by a greater emphasis on HIV care per se and the risk of these patients to develop opportunistic infections followed by a lack of attention to other more common non-communicable diseases (NCD). Nonetheless, a comprehensive and integrative approach for the care of general and specific populations is urgently required^24^.

Several studies have described possible unified models of NCD and HIV care and treatment in low-income countries^25^. Implementation feasibility of the proposed models should consider the individual characteristics of health care services. The possible approaches vary from integration of NCD services into HIV care centers to integration of HIV care into primary health care with NCD services and simultaneous integration of both services^25^. Several measures are to be adopted for successful implementation of each proposed model. However, regardless of the selected model, concerns with respect to human and structural resources, supply chains, and patient education are required to be addressed, with a special focus on early screening, diagnosis, and concomitant effective care.

This study has some limitations. For instance, the inclusion of PLHIV who visited the outpatient clinic may have resulted in the inadequate estimation of the number of hypertensive and normotensive individuals in the general population, leading to the misperception that these results truly reflect the local community. Nonetheless, these results reflect the actual practice patterns and enable a more precise conclusion regarding real-life scenarios in countries with similar economic development and public health care challenges as Brazil.

In conclusion, controlling hypertension is important to minimize CVDs and CVD-related mortality among PLHIV. An increased prevalence of hypertensive status was found in PLHIV, with most of them unaware of it. In addition, several gaps in the hypertension care cascade demonstrated a poor approach to the problem, which should be addressed through a multidisciplinary approach at different levels of public health care. Therefore, an improvement in screening, diagnosis, treatment, and control is urgently required. The provision of highly comprehensive services for the care and treatment of NCDs and HIV is key.
